# Biosynthesis and Characterization of Polyhydroxyalkanoates Copolymers Produced by *Pseudomonas putida* Bet001 Isolated from Palm Oil Mill Effluent

**DOI:** 10.1371/journal.pone.0045214

**Published:** 2012-09-20

**Authors:** Ahmad Mohammed Gumel, Mohamad Suffian Mohamad Annuar, Thorsten Heidelberg

**Affiliations:** 1 Institute of Biological Sciences, Faculty of Science, University of Malaya, Kuala Lumpur, Malaysia; 2 Department of Chemistry, Faculty of Science, University of Malaya, Kuala Lumpur, Malaysia; Missouri University of Science and Technology, United States of America

## Abstract

The biosynthesis and characterization of medium chain length poly-3-hydroxyalkanoates (mcl-PHA) produced by *Pseudomonas putida* Bet001 isolated from palm oil mill effluent was studied. The biosynthesis of mcl-PHA in this newly isolated microorganism follows a growth-associated trend. Mcl-PHA accumulation ranging from 49.7 to 68.9% on cell dry weight (CDW) basis were observed when fatty acids ranging from octanoic acid (C_8∶0_) to oleic acid (C_18∶1_) were used as sole carbon and energy source. Molecular weight of the polymer was found to be ranging from 55.7 to 77.7 kDa. Depending on the type of fatty acid used, the ^1^H NMR and GCMSMS analyses of the chiral polymer showed a composition of even and odd carbon atom chain with monomer length of C4 to C14 with C8 and C10 as the principal monomers. No unsaturated monomer was detected. Thermo-chemical analyses showed the accumulated PHA to be semi-crystalline polymer with good thermal stability, having a thermal degradation temperature (*T*_d_) of 264.6 to 318.8 (±0.2) ^o^C, melting temperature (*T*_m_) of 43. (±0.2) ^o^C, glass transition temperature (*T*_g_) of −1.0 (±0.2) ^o^C and apparent melting enthalpy of fusion (Δ*H*_f_) of 100.9 (±0.1) J g^−1^.

## Introduction

The current increase in the utilization of polyhydroxyalkanoates (PHAs) in various industrial and biomedical applications is due to their biodegradability, compatibility, resorbability and piezoelectricity [1]. Their diverse chemical properties have made them a subject of many research interests. In fact, the diversity of different monomeric components in accumulated PHA that arises from superb biological polymerization system resulted in potentially diversified high-molecular weight polymeric materials. Growing concern over environmental pollution has heightened the interest in these biodegradable polymers over their chemically synthesized counterparts.

The low level of medium-chain-length PHA (mcl-PHA) and limited substrate utilization in different bacterial species, poses limitation to industrial production of these polymers [2]. The increasing demand of highly functionalized PHA for specialty applications, warrant the bio-prospecting of bacterial species capable of accumulating these biodegradable polymers. Several bacterial species known to accumulate PHA have been isolated from different ecological niches [3], [4], [5], [6], [7], [8]. Among the extensively studied bacterial species are those belonging to genus *Pseudomonas* [9], [10], [11], [12], [13], that are known to accumulate intracellular PHA under limited cell growth phase in the presence of abundant carbon source and minimal nutrient condition [14]. When fatty acid is fed to these bacteria, it passes through beta-oxidation biosynthetic pathway to produce the PHA intermediates thereby losing two (2) carbon atoms per each cycle [15], [16].

A number of published works use unsaturated fatty acids such as oleic acid (C_18∶1_) to produce mcl-PHA with unsaturated side-chain monomers [17], [18], [19], [20]. In this study, the growth and biosynthesis of mcl-PHA by a strain of *Pseudomonas putida* isolated from palm oil mill effluent are reported, along with characterization of the mcl-PHA copolymer produced when the bacterium is fed with oleic acid (C_18∶1_). For comparison, selected fatty acids *viz.* octanoic acid (C_8∶0_), dodecanoic acid (C_12∶0_) and hexadecanoic acid (C_16∶0_) were also tested as substrates.

## Methods

### Bacterial Isolation and Characterization

*Pseudomonas putida* Bet001 was isolated from palm oil mill effluent using isolation agar medium containing (g L^−1^): peptone 20; K_2_SO_4_ 10; MgCl_2_.6H_2_O 1.4; irgasan 0.025; glycerol 25.2 and agar 13.6. Preliminary biochemical characterization was done using api® 20 NE (bioMerieux® USA) biochemical typing kits as stated in manufacturer’s guideline. Intracellular PHA accumulation was screened using both Sudan Black B and Nile red staining techniques according to literatures [21], [22]. Intracellular PHA granules accumulation was also observed using transmission electron microscopy (Philips CM12, United Kingdom). The morphology of the bacterial colonies isolated and the Gram staining characteristics were observed under Motic® microscope BA200 equipped with Moticam 2300 camera (Motic®, Japan). GF1 DNA extraction kit (Vivantis Sdn Bhd, Malaysia) was used to extract the bacterial DNA for the 16S rRNA molecular characterization according to manufacturer’s guidelines. Post PCR analysis was done by blasting of both forward and reverse sequences against sequences in GeneBank (NCBI) and RDPII (Ribosomal Database Project II) databases to search for homology sequences. The extracted DNA nucleotide sequence has been deposited in European Nucleotide Archive (ENA) with accession number HE573173.

### Culture Conditions and Fermentation Process

Sterile growth medium (100 ml per each 250 ml conical flask) containing (g L^−1^): yeast extract 10.0 (Bacto™ USA), nutrient broth 15.0 (Merck, Germany) and ammonium sulfate 5.0 (Sigma Aldrich, Germany) was used as inoculum cultivation medium for the bacterium. 3% (v/v) of *P. putida* Bet001 stock inoculum was introduced into this medium aseptically and incubated in shaking incubator (Daihan LabTech®, Korea) at 30°C, 250 rpm for 24 hours. The biomass was harvested from 3 ml culture broth *via* centrifugation at 4°C, 9000 ×*g* for 10 min. The recovered biomass was seeded aseptically into PHA production medium. The medium contains sterile minerals of (g L^−1^) sodium ammonium hydrogen phosphate tetrahydrate [NaNH_4_HPO_4_.4H_2_O] 3.5, K_2_HPO_4_ 5.7, KH_2_PO_4_ 3.7, and 10 mM oleic acid (unless otherwise stated) at a pH of 7.0 (±0.1). Separate sterile solutions of 0.1 M MgSO_4_.7H_2_O and trace elements (MT) containing (g L^−1^): CaCl_2_.2H_2_O 1.47, CoCl_2_.6H_2_O 2.38, CuCl_2_.2H_2_O 0.17, FeSO_4_.7H_2_O 2.78, MnCl_2_.4H_2_O 1.98 and ZnSO_4_.7H_2_O 0.29 dissolved in 1 M HCl were aseptically added to the PHA production media prior to inoculation at 1.0% (v/v) and 0.1% (v/v), respectively. 5 g L^−1^ of harvested biomass from inoculum growth media was seeded into this medium (100 ml per 250 ml conical flask) aseptically and incubated as described previously for a total period of 48 hours unless otherwise stated. PHA extraction was done according to literature [10]. 5 g of vacuum dried biomass was suspended in 30 ml chloroform and refluxed at 60°C for 4 hours. The refluxed chloroform containing the extracted PHA was filtered out of the residual biomass debris using Buchner filter funnel equipped with sintered glass. The filtrate was then concentrated under vacuum using rotary evaporator at 40°C until about a fifth of the total volume. The polymer was precipitated out of the concentrate by dispersing it into a beaker containing excess cold methanol (at least 1∶4 volume ratio). The mcl-PHA obtained underwent a minimum of three-repeated purification process by sequential chloroform dissolution and methanol re-precipitation.

### Biomass Estimation

A known biomass dry weight concentrations were used to prepare a standard calibration, using Jasco V-630 UV/VIS spectrophotometer (Jasco, Japan) at the absorbance of 600 nm. Aliquote samples were withdrawn aseptically at regular intervals and centrifuged at 9000 ×*g* for 10 min, then washed twice with 0.05 M phosphate buffer. After final centrifugation, the cells were re-suspended in the same buffer prior to absorbance reading against the phosphate buffer blank.

### Residual Fatty Acid Quantification

The residual fatty acid estimation was done according to Marseno et al. [23] modified protocol. Cell free supernatant (1 ml) was mixed with *n*-heptane (3 ml) and centrifuge at 9000 × *g* to separate the residual fatty acid. 2 ml of the top layer of *n*-heptane was withdrawn into a test tube, to this solution, 200 µl of 5% (w/v) copper (II) acetate monohydrate solution (5 g copper (II) acetate monohydrate dissolved in 90 ml distilled water; pyridine and distilled water were respectively used to adjust the solution pH to 6 and bring the final volume to100 ml) was added and vortexed for 60 sec. This mixture was allowed to stand for 20 sec before it was read at 705 nm against distilled water blank using Jasco V-630 UV/VIS spectrophotometer (Jasco, Japan). A standard calibration was performed using a stock of different fatty acid concentrations (0.5 to 1 mM) that were made by dissolving the specified mass of fatty acid in *n*-heptane and treated as mentioned earlier.

### Residual Ammonium Quantification

Residual ammonium was quantified using phenol hypochlorite method as described by Solorzano [24]. Cell free supernatant (2 ml) was diluted with distilled water to 5 ml. To this solution 0.2 ml of alcoholic phenol (10 g phenol in 90 ml absolute ethanol) was added followed by the addition of 0.2 ml of sodium nitroprusside solution (0.5 g sodium nitroprusside in 99.5 ml distilled water). Oxidizing solution (0.5 ml) was added to the mixture while agitating to ensure uniform indole-phenol coloration. The oxidizing solution was composed of 4∶1 mixture of alkaline solution to oxidizing reagent. The alkaline solution was composed of 100 g trisodium citrate, 5 g sodium hydroxide dissolved in 500 ml of distilled water. The oxidizing reagent was 1.5 N sodium hypochlorite solution. The mixture was then allowed to stand for an hour at room temperature (27°C) resulting in light-blue coloration that was measured as described above at 640 nm against blank distilled water. Residual ammonium concentration was then quantified in reference to standard calibration curve of known ammonium concentrations.

### PHA Authentication

#### FTIR spectroscopy

Attenuated total reflectance (ATR) FTIR was used to record the PHA spectrum on Perkin-Elmer FTIR *spectrum-400* spectrometer (Perkin-Elmer Inc., Wellesley, MA, USA) at room temperature (27°C) and a scan range of 400 to 4000 cm^−1^ was applied for 10 scans. A paste of 0.01 g of the sample was prepared in dichloromethane. A liquid film of the mixture was then applied on to NaCl crystal window and the spectrum was read after the solvent has been dried.

#### Proton NMR analysis

JEOL JNM-GSX 270 FT-NMR (JOEL Ltd, Tokyo, Japan) spectrometer was used to record the ^1^H NMR spectrum at 250 MHz against tetramethylsilane (TMS) internal reference standard. Approximately 5 mg PHA sample was dissolved in 2 ml deuterated chloroform (CDCl_3_) and filtered into NMR tube using borosilicate glass syringe equipped with 0.22 µm PTFE disposable filter (11807–25, Sartorius Stedim, Germany).

#### GC-MSMS analysis

GC-MSMS was recorded on Agilent triple quadrupole 7000B (Agilent, USA), equipped with GCMSMS triple axis detector carrying Agilent HP-5 ms column (30 m length× 0.25 mm internal diameter× 0.25 µm film). PHA sample in the form of hydroxyalkanoic acids methyl esters were used for the GC-MSMS analysis according to method reported in literatures [25], [26]. Methanolyzed sample (1 µl) was automatically injected into the GC at a split ratio of 1∶50. The injection temperature was set at 280°C while the oven and column temperatures were programmed as 40°C for 1 min then increase to 120°C at 15°C min^−1^, hold for 2 min, and increase to 250°C at 10°C min^−1^ hold for 15 min. Helium was used as carrier gas at 48.3 ml min^−1^ and 0.41 bar pressure. Mass spectra were acquired at 1250 scan speed using electron impact energy of 70 eV at 200°C ion-source and 280^o^C interface temperatures respectively. Standard monomers of methyl hydroxyalkanoates (Larodan, Sweden) were used as reference for peak retention time and ionization mass determination.

#### Gel permeation chromatography (GPC)

Waters 600 (Waters Corp, Milford, MA, USA) equipped with a Waters refractive index detector (model 2414) having the following gel columns (7.8 mm internal diameter 300 mm) in series: HR1, HR2, HR5E and HR5E Waters Styragel HR-THF was used to record the chromatogram relative to calibration curve of standard monodisperse polystyrenes (3.72 x10^2^, 2.63×10^3^, 9.10×10^3^, 3.79×10^4^, 3.55×10^5^, 7.06×10^5^, 3.84×10^6^ and 6.77×10^6^ Da). The PHA samples were dissolved in tetrahydrofuran (THF) at a concentration of 2.0 mg mL^−1^, filtered through a 0.22 µm PTFE filter. 100 µL aliquot of the sample was injected at 40°C using THF as a mobile phase at a flow rate of 1.0 mL min^−1^.

### Thermal Analyses

#### Differential scanning calorimetry (DSC)

The extracted PHA was subjected to differential thermal analysis using Mettler-Toledo differential scanning calorimeter (DSC 822e; Mettler-Toledo, USA) running STARe DSC ver 8.10 software; equipped with HAAKE EK90/MT digital immersion cooler (Thermo Fischer Scientific, USA). Scans were made at a temperature range of −60°C to 180°C with a heating rate of 10°C min^−1^ under a nitrogen flow rate of 0.12 L min^−1^at a head pressure of 1.5 bar. The melting temperature (*T*_m_) was taken at the endothermic peak of the DSC thermogram. The endothermic melting enthalpy (Δ*H*_m_) was used to calculate the polymer crystallinity (*X*_p_) on the basis of melting enthalpy (Δ*H*_m_^o^) of 100% crystalline PHB according to equation 1 as reported somewhere else [27], assuming 142 J g^−1^ as the melting enthalpy of 100% crystalline PHB as cited in literature [28].
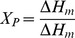
(1)


#### Thermogravimetric analysis (TGA)

TGA analysis was performed on a Perkin-Elmer TGA 4000 instrument. The sample was heated from 50°C to 900°C at a rate of 10°C min^−1^ under a nitrogen flow rate of 20 ml min^−1^.

## Results and Discussion

### Strain Characterization and PHA Production

**Figure 1:**
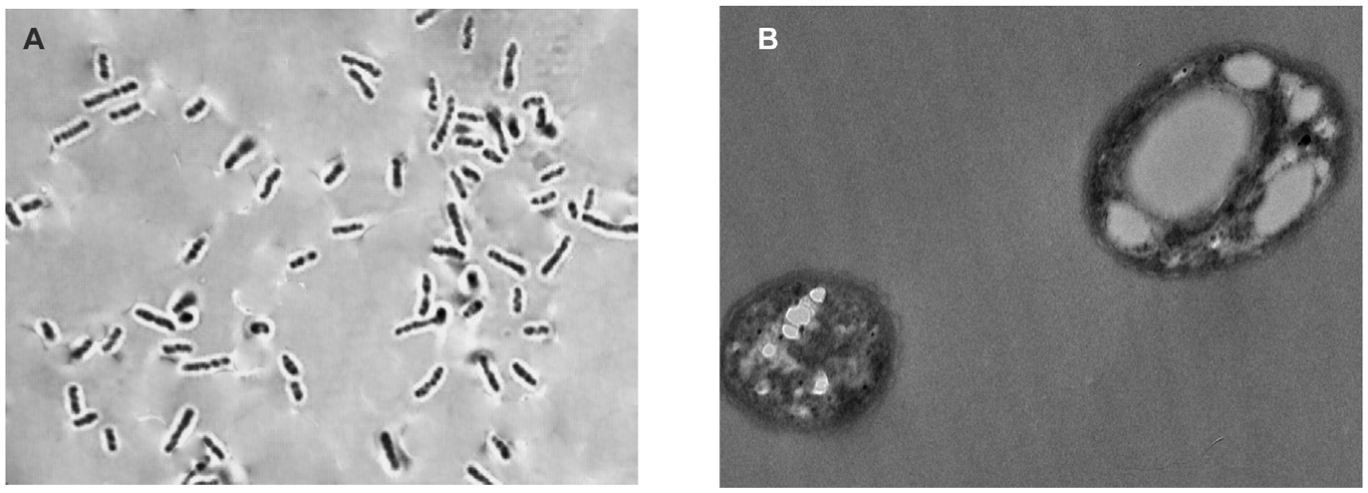
*Pseudomonas putida* Bet001 cells showing PHA inclusion. (A) phase contrast (X 100 magnification) and (B) T.E.M (X 5000 magnification) when cultivated on palmitic acid (C_16∶0_) at 30°C, 200 rpm for 48 h.

**Figure 2:**
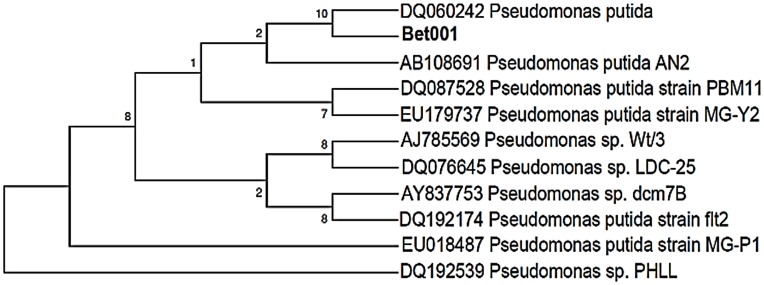
Neighbor joining phylogenetic tree showing the interrelationship between isolate Bet001 and top 10 Blast hits from RDP database.

Sudan Black B staining was used in the microscopic observation for a possible accumulation of intracellular PHA by the isolated strain (Fig. 1a). Further confirmation of the presence of accumulated granule was made by transmission electron microscopy (Fig. 1b). The bacterial strain was further characterized using biochemical analyses and 16S rRNA molecular characterization. The biochemical analyses indicated a probability of 99.5% for the isolate to be identified as *Pseudomonas putida*. This result was corroborated by RDP blast neighbor-joining phylogenetic analysis (Fig. 2), where 99% analogy was observed between the isolate and *Pseudomonas putida* strains AJ785569, AN2, BCNU106, DQ060242 and DQ087528. This isolate was designated as *P. putida* Bet001and subsequently used in the PHA production using shake flask in a two-step batch fermentation process with different fatty acids as a sole carbon and energy source. PHA accumulation was observed to span from 49.7 to 68.9 dry weight % and was composed of varied monomer fractions ranging from 3-hydroxyhexanoate to 3-hydroxytetradecanoate depending on the culture conditions and the type of fatty acids used.

### PHA Authentication

#### FTIR spectroscopy

**Figure 3:**
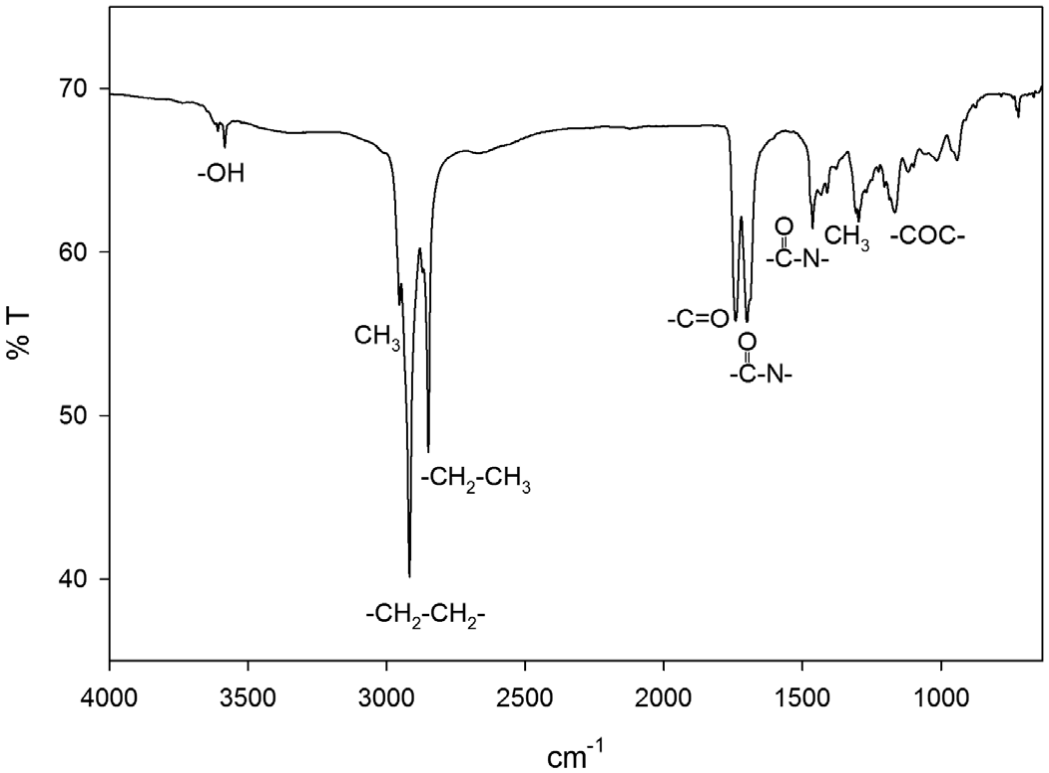
FTIR-ATR spectrum of the PHA extracted from *P. putida* Bet001 fed on oleic acid (C_18∶1_).

The extracted PHA sample was subjected to nondestructive attenuated total reflectance FT-IR. Observed infrared absorption at 3420.20 cm^−1^ was assigned to the hydroxyl group of the polymer chain [29]. Absorption band at 2955.76 cm^−1^ was assigned to asymmetric methyl group. Asymmetric CH_2_ of the lateral monomeric chains were assigned to the stretching vibration at 2925.98 cm^−1^. The absorption at 2855.99 cm^−1^ has been assigned to symmetrical CH_3_ and the intensity of the band has been reported to be due to conformational disorder obtained in the process of crystallization [28]. While the absorption band of 1741.44 cm^−1^ has been reported to be a PHA marker band assigned to carbonyl (C = O) ester bond stretching vibration according to Randriamahefa et al. [30]. The vibrations at 1659.50 and 1460.39 cm^−1^ have been assigned to bacterial intracellular amide (–CO–N–) I and II respectively (Fig. 3); furthermore, these bands were also reported to be species specific [30]. Absorption at 1378.83 cm^−1^ is assigned to terminal CH_3_ groups [12], while that at 1259.89 cm^−1^ is due to asymmetric C–O–C stretching vibration. Series of absorption bands at 1166.87 cm^−1^ to 619.39 cm^−1^ were assigned to C–O and C–C stretching vibration in the amorphous phase.

#### PHA proton NMR characterization

**Figure 4:**
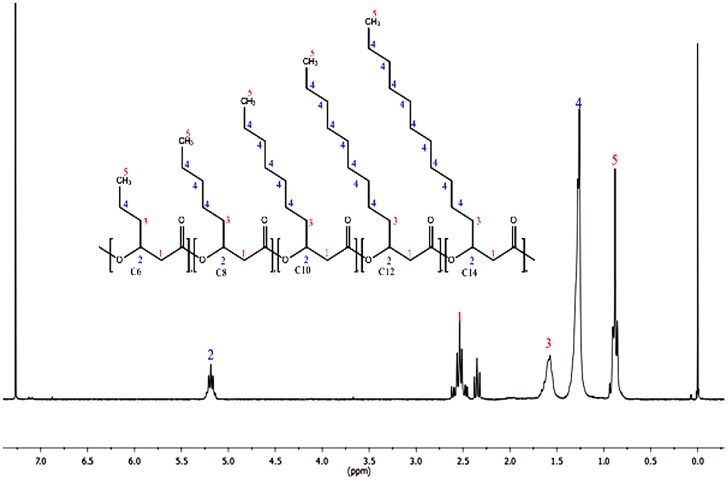
NMR (^1^H) spectrum of the PHA extracted from *P. putida* Bet001 fed on palmitic acid (C_16∶0_).

The proton NMR spectrum of the produced PHA was characterized in reference to the internal standard trimethylsilane. The observed multiplet peaks number 1(δ 2.5 ppm) and triplet peaks 2 (δ 5.2 ppm) are assigned to methylene and methine protons of the α and β-carbon respectively [15], [16], [18], [31]. Contrary to some literatures assigning the observed triplets at chemical shift 2.3 ppm (Fig. 4) to unsaturation of PHA monomers [18], [26], upon further analysis using GCMSMS, we proposed the peak to be due to hydrophobic oleate and palmitate from cellular membrane lipids. Peak number 3 (δ 1.6 ppm) is assigned to methylene protons adjacent to the β-carbon in the side-chains [15], [16], [18], [31]. Peak number 4 (δ 1.3) and triplet number 5 (δ 0.9) are assigned to the methylene protons and terminal methyl proton of the side-chains respectively [15], [16], [18], [31].

### Thermal Analyses

The calorimetric scan of the extracted PHA showed the endothermic melting temperature (*T*_m_) of this polymer to be 43.0 (±0.2) ^o^C, glass transition temperature (*T*_g_) of −1.0 (±0.2) ^o^C and apparent melting enthalpy of fusion (Δ*H*_f_) of 100.9 (±0.1) J g^−1^. The relatively low melting and glass transition temperatures were attributed to the random composition of 3HA monomers in the polymer (see later discussion). This observation was found to be in good agreement with what has been reported by Matsusaki et al. [32] when observing the PHA biosynthesis in recombinant *Pseudomonas sp*. 6 1–3 strain fed with dodecanoic acid (C_12∶0_). The bacterium produced random copolymer of PHA with endothermic melting temperature of 42°C. The observed high apparent heat of fusion could be due to the presence of long chain monomer fraction which in turn was reported to favor side-chain crystallization resulting in polymer with high apparent enthalpy of fusion. This observation was found to agree with reported literatures [15], [16], [33].

PHA thermal stability is an important parameter to be studied during polymer analyses as it reflects the maximum temperature the polymer can withstand before thermal decomposition. Thermal gravimetric analysis of the extracted polymer showed an increase in polymer thermal stability with increasing fatty acid chain length fed i.e. from C_8∶0_ to C_18∶1_ (thermal degradation temperature of 264.6 to 318.8^o^C ±0.2).This is attributed to the increase in the fractions of longer monomer such as 3HDD and 3HTD that became incorporated into the PHA from the metabolism of the longer fatty acid substrates (Table 1). It has been reported also that this increase in thermal stability could be due to random co-monomer chain-length that resulted in high side chain crystallization conferring thermal stability to the polymer [15], [33].

**Table 1:** PHA composition as a function of carbon source (max. standard error ±5%).

Carbon	%PHA	PHA mole fraction (mole %)							*M*_n_	*M*_w_	PDI
**source**	**(w/w)**										
		3HB†	3HHx‡	3HHp∥	3HO 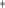	3HD#	3HDD§	3HTD¶			
		(C4)	(C6)	(C7)	(C8)	(C10)	(C12)	(C14)			
**C_8∶0_**	49.7	ND	8.1	ND	76.2	11.0	4.7	ND	38685	77657	2.0
**C_12∶0_**	54.5	ND	3.5	ND	38.2	38.9	19.4	ND	31388	60056	1.9
**C_16∶0_**	65.3	ND	4.1	ND	36.9	34.8	18.0	6.3	13608	55685	4.1
**C_18∶1_**	68.9	1.5	5.0	0.7	31.8	24.1	22.9	14.1	35308	74958	2.1

† (hydroxybutyrate)

‡ (hydroxyhexanoate)

∥ (hydroxyheptanoate)

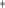
(hydroxyoctanoate)

# (hydroxydecanoate)

§ (hydroxydodecanoate)

¶ (hydroxytetradecanoate), ND (not detected).

### Effect of Carbon Source on the PHA Composition and Yield

The effect of different carbon source on PHA composition has been studied. The composition of PHA accumulated by *P. putida* Bet001 grown on different carbon sources at 30°C for 48 hours is presented in Table 1. PHA accumulation in the range of 49.7 to 68.9% (w/w) has been observed when the organism is fed with C_8∶0_ to C_18∶1_ fatty acids, with a polymer composition ranging from 3HB (four carbon atom length) to 3HTD (fourteen carbon atom length) except when oleic acid was used as substrate, a small amount of odd chain monomer i.e. 3HHp (seven carbon atom length) was also detected. At first, this was considered as possible contamination of the extracted polymer sample by cellular components. Thus, the purification step was repeated at least three times and the polymer re-extracted for GCMSMS analysis. The persistence of the strong signal detected by mass spectrometry for the presence of 3HHp entails re-consideration of the contamination hypothesis with non-PHA components. From the basic knowledge about β-oxidation of even carbon atom length of fatty acids, it is not clear at this point the exact reason for the incorporation of an odd carbon atom length monomer like 3HHp into the polymer when oleic acid (C_18∶1_) was used as a sole carbon and energy source for the bacterial growth and PHA accumulation, and not with other fatty acids tested.

Based on mass spectrometric analyses, feeding dodecanoic acid as sole carbon source resulted in PHA composed of four different monomers (3HHx 3.54 mole%, 3HO 38.19 mole%, 3HD 38.85 mole% and 3HDD 19.42 mole%). Similarly, Chung et al. [15] reported *P. entomophila* L48 to accumulate PHA having four different monomer units when fed with dodecanoic acid, with monomer fractions of 3HD (38.6%), 3HO (44.5%) as the dominant fractions. Feeding hexadecanoic acid as a carbon and energy source to *P. putida* Bet001 resulted in PHA with five different monomers (3HHx 4.06 mole%, 3HO 36.93 mole%, 3HD 34.78 mole%, 3HDD 17.97 mole% and 3HTD 6.26 mole%). When oleic acid (C18∶1) was fed under the same conditions, copolymer with up to seven different monomers of 3HB (1.52 mole%), 3HHx (5.01 mole%), 3HO (31.81 mole%), 3HD (24.07 mole%), 3HDD (22.86 mole%), 3HTD (14.05 mole%) and a small amount of odd carbon chain length monomer of 3-hydroxyheptanoate (3HHp) (0.68 mole mole%) were obtained. In contrast to other fatty acids tested, when oleic acid was fed, a short-chain length monomer of 3HB (four carbon atom length) accumulated. The accumulation of 3HB monomer was not detected in the bacterium when fatty acids C_8∶0_, C_12∶0_ and C_16∶0_ were fed. Again, similar to the accumulation of 3HHp, the exact reason for this observation is not clear at this stage. It was also observed that when oleic acid (C_18∶1_) was fed, no unsaturated PHA monomer was detected in the polymer produced. The most abundant monomers were C8 and C10 in all the samples tested, and this is in agreement with the hypothesis that PHA formed by Pseudomonads belonging to rRNA homology group I have C8 and C10 monomers as dominant components when grown on C8 to C18 fatty acids substrate [18], [34].

From the results presented in Table 1, the relationship between the PHA composition and the structure of the carbon source substrate showed that the PHA biosynthetic precursors in this bacterium are very likely fed by β-oxidation pathway.

### Effect of Nitrogen Source Concentration on Specific Growth Rate *(µ)* and PHA Yield

**Figure 5:**
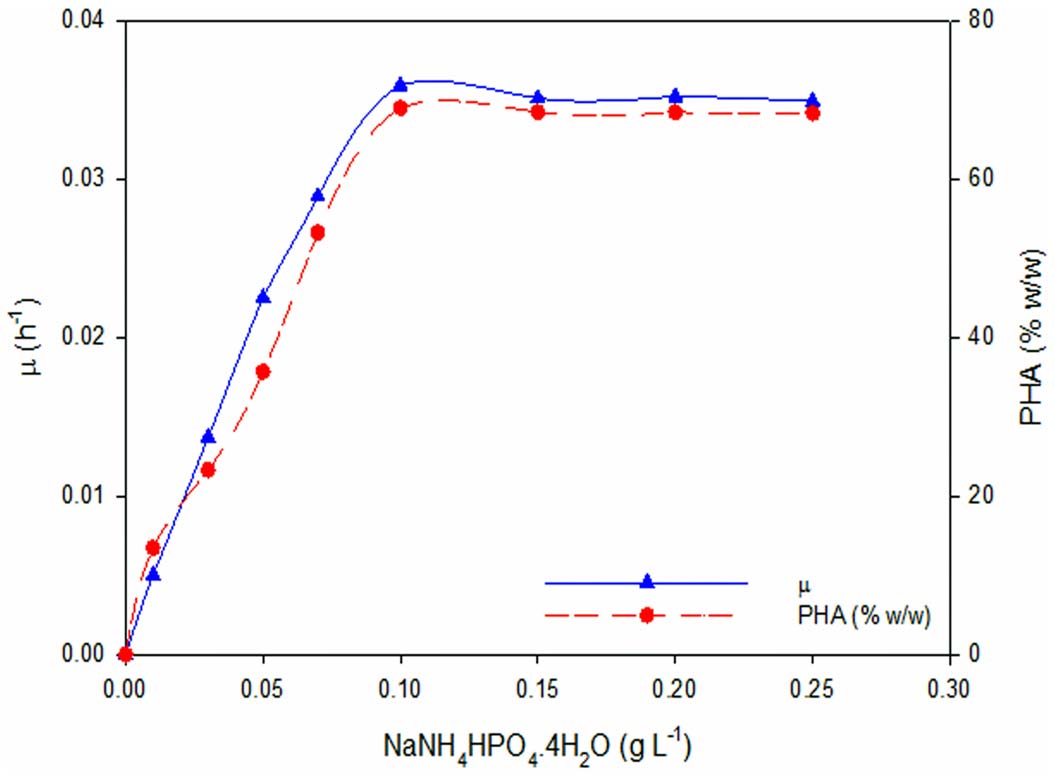
Specific growth rate and PHA content as a function of ammonium ion concentrations in *P. putida* Bet001 fed on oleic acid (C_18∶1_).

The effect of nitrogen source concentration on specific growth rate and PHA yield during PHA biosynthesis in *P. putida* Bet001 is depicted in Fig. 5. It is clear that as the ammonium ion concentration increases from 0.01 to 0.1 g L^−1^, the specific growth (*µ*) also increases from 0.005 to 0.036 h^−1^with corresponding PHA yield of 13.4 to 68.9% (w/w) respectively. Increasing nitrogen source beyond this point resulted in a gradual plateau in the specific growth rate, probably due to the carbon source concentration began to be limited as in this study where the ammonium ion concentration was varied and the carbon source concentration was kept fixed. In a similar study, Annuar et al. [35] observed the increase in specific growth rate of *P. putida* PGA1 from 0 to 0.18 h^−1^ by increasing the ammonium ion concentration from 0 to 0.1 g L^−1^; further increase in ammonium ion concentration (0.6 to 0.8 g L^−1^) resulted in a decrease in the specific growth rate to 0.14 h^−1^. Interestingly, the PHA fraction from the total biomass also increases as the specific growth rate increases, indicating a growth associated PHA biosynthesis in this bacterium. The PHA fraction reached a constant value when specific growth rate became constant at high ammonium ion concentrations. This is in contrast with the recognized PHA accumulation in many *Pseudomonas* species, where the highest accumulation is observed when the specific growth rate of the organism is low under nutrient limitation/starvation [14], [18], [35]. The observed increase in the specific growth rate at low ammonium ion concentrations (≤0.1 g L^−1^) could probably be due to high ammonium uptake by the cells [35].

### Influence of Carbon-nitrogen Molar Ratio on Biomass and Polymer Content

It has been observed that PHA content of the cell can be increased by changing the molar ratio between carbon-to-nitrogen (C:N) source (Table 2). By fixing the ammonium ion concentration at 0.1 g L^−1^ while varying the carbon source concentration; it is observed that increase in C:N molar ratio from 10 to 30 was followed by increase in percentage PHA accumulation and biomass from 16 to 69% and from 9.6 to 15.3 g L^−1^, respectively. The proportional increase in biomass with increase of C:N ratio up to 20, could indicate that below 20 carbon is limiting growth. Additional increase in C:N molar ratio after 20 however, did not results in further increase in biomass amount, as it is expected that the ammonium concentration no longer sufficient to sustain extended growth. The results from this experiment offer additional support for the growth-linked PHA accumulation in this bacterium. Maximum PHA accumulation at approximately 70% was found for C:N molar ratios of 25 and 30. The molecular weight of the PHA from the different C:N molar ratios tested fell within a narrow range of 37 to 75 kDa. The crystallinity (*X*_p_) and poly dispersity index (PDI) remained constant at all the C:N molar ratio tested.

**Table 2:** Effect of carbon-to-nitrogen molar ratio on PHA content and biomass in batch culture of *P. putida* Bet001grown on oleic acid (C_18∶1_) (max. standard error ±5%).

C:N	PHA	Biomass	*M*_w_	*X*_p_	*PDI*
(mole)	(% w/w dried biomass)	(g L^−1^)	(Da)		
10	16.2	9.60	49147	0.76	2.1
15	39.3	11.4	52143	0.74	2.1
20	55.5	15.3	74958	0.72	2.1
25	68.9	14.5	45678	0.75	2.1
30	68.4	13.9	37444	0.73	2.2

### Conclusions

A chiral PHA copolymer consisting of monomers ranging from 3-hydroxybutyrate (3HB) to 3-hydroxytetradecanoate (3HTD) were synthesized by a new isolate *P. putida* Bet001 when fed with different fatty acids. The biosynthesis of the PHA by this new isolate is growth-associated. When fed with oleic acid (C_18∶1_), the PHA extracted from the isolate showed the presence of a monomer with odd carbon atom length i.e. 3-hydroxyheptanoate (3HHp). However, no unsaturated monomer was detected when oleic acid (C_18∶1_) was fed. *P. putida* Bet001 accumulated about 69% PHA on cell dry weight basis with good thermal stability, relatively high molecular weight and crystallinity.
